# Effect of nephrostomy sheath size on renal pelvic pressure during endoscopic combined intrarenal surgery: artificial kidney model study

**DOI:** 10.1186/s12894-024-01458-z

**Published:** 2024-04-03

**Authors:** Takashi Iguchi, Shimpei Yamashita, Yasuo Kohjimoto, Tadashi Tanioku, Tomoyuki Kawamata, Isao Hara

**Affiliations:** 1 Department of Urology, Wakayama Medical University Hospital, 811-1 Kimiidera, 641-8509 Wakayama, Japan; 2 Department of Anesthesiology, Wakayama Medical University Hospital, Wakayama, Japan

**Keywords:** Lithotripsy, Nephroscope, ECIRS, Renal pelvic pressure, Urinary tract infection

## Abstract

**Background:**

This study aimed to evaluate the intrarenal pelvic pressure in endoscopic combined intrarenal surgery using an artificial kidney model.

**Methods:**

An artificial kidney model was created using the Urovac evacuator™. Four sizes of nephrostomy sheaths (MIP-L: 25/26 Fr, MIP-M: 16.5/17.5 Fr, MIP-S: 11/12 Fr, MIP-XS: 8.5/9.5 Fr) and two sizes of ureteral access sheaths (12/14 Fr and 10/12 Fr) were installed into the model. For each combination of nephrostomy and ureteral access sheath, renal pelvic pressure was measured with and without insertion of the retrograde flexible ureteroscope. Irrigation from the nephroscope was adjusted to 40–160 mmHg using an automatic irrigation device, and the irrigation of the ureteroscope was by spontaneous dripping at 80 cmH_2_O. Conditions were measured six times, and the renal pelvic pressure was compared in different conditions.

**Results:**

Without ureteroscope insertion through the ureteral access sheath, the renal pelvic pressure never exceeded 30 mmHg. Meanwhile, when the ureteroscope was inserted, the renal pelvic pressure increased as the nephrostomy sheath and ureteral access sheath became narrower and as the irrigation pressure increased. Intrarenal pelvic pressure exceeded 30 mmHg when the irrigation pressure was increased in 12/14 Fr ureteral access sheath when MIP-XS was used, and in 10/12 Fr ureteral access sheath when MIP-XS and MIP-S were used.

**Conclusions:**

The use of a thin nephrostomy sheath in endoscopic combined intrarenal surgery can lead to increased intrarenal pelvic pressure. Although our results are from an artificial kidney model, special care is suggested to be required when using a retrograde flexible ureteroscope simultaneously in treatment of patients.

**Supplementary Information:**

The online version contains supplementary material available at 10.1186/s12894-024-01458-z.

## Background

Treatment of urinary tract stones has become a major field, with dramatic advances in surgical techniques and in the development of endoscopic instruments. In addition to research on surgical techniques such as lithotripsy and extraction, there is wide research into intraoperative and postoperative complications. One such complication is the effect on the kidney due to increased renal pelvic pressure [1]. In upper urinary tract endourology, irrigate inflow and drainage are thought to be important because they affect renal pelvic pressure [2].

Studies have shown that the renal pelvic pressure is 7.5–14.7 cmH_2_O under physiological conditions and increases to 7.5–25 cmH_2_O due to diuretic action [3, 4]. In an experiment that examined the effects of increased renal pelvic pressure on the body, intrarenal reflux was induced at a pressure of approximately 40 cmH_2_O (30 mmHg) in porcine and human kidneys [1, 5]. Increased renal pelvic pressure can lead to intrarenal backflow and even rupture of the collecting system [6, 7]. This may result from direct vascular reabsorption of extravasated fluid. Possible complications include sepsis due to bacterial and endotoxin blood transfer and potential renal damage at high irrigation pressures [8, 9].

Percutaneous nephrolithotomy (PCNL) is considered to be a major surgical technique that is mainly used for large kidney stones [10, 11]. Renal pelvic pressure measurements using the PCNL device have been evaluated in several reports. However, there are few reports that evaluated renal pelvic pressure with endoscopic combined intrarenal surgery (ECIRS). Importantly, this study is the first to examine and made comparison using an ultra-thin device with a diameter of ≤ 10 Fr. To evaluate renal pelvic pressure in ECIRS, we therefore created an artificial kidney model and evaluated it under various conditions.

## Methods

### Model (Fig. 1)

An artificial kidney model was created by using Urovac evacuator™ (Boston Scientific, Marlborough, MA, USA; capacity: 340 ml, length: 14 cm, maximum diameter: 7.3 cm, thickness: 3.2 mm) as a renal pelvis calyx based on previous studies [12, 13]. The nephrostomy sheath was connected to the Urovac evacuator™ so that it was 70° above the horizontal plane, which was designed to represent puncture of the lower calyx in a prone position in a human body. A silicon connector was attached so that the nephrostomy sheath could be replaced. A ureteral access sheath (UAS) was installed at a site that resembled the renal pelvic ureteral junction, and it was made possible to replace it with a silicone connector. An arterial tonometry line (Edward Phillips, USA) was attached to the head side of Urovac evacuator™, and the renal pelvic pressure was measured. The position of the transducer was adjusted to the height of the base of the Urovac evacuator™, and zero correction was performed in any of the measurements.

**Fig. 1:**
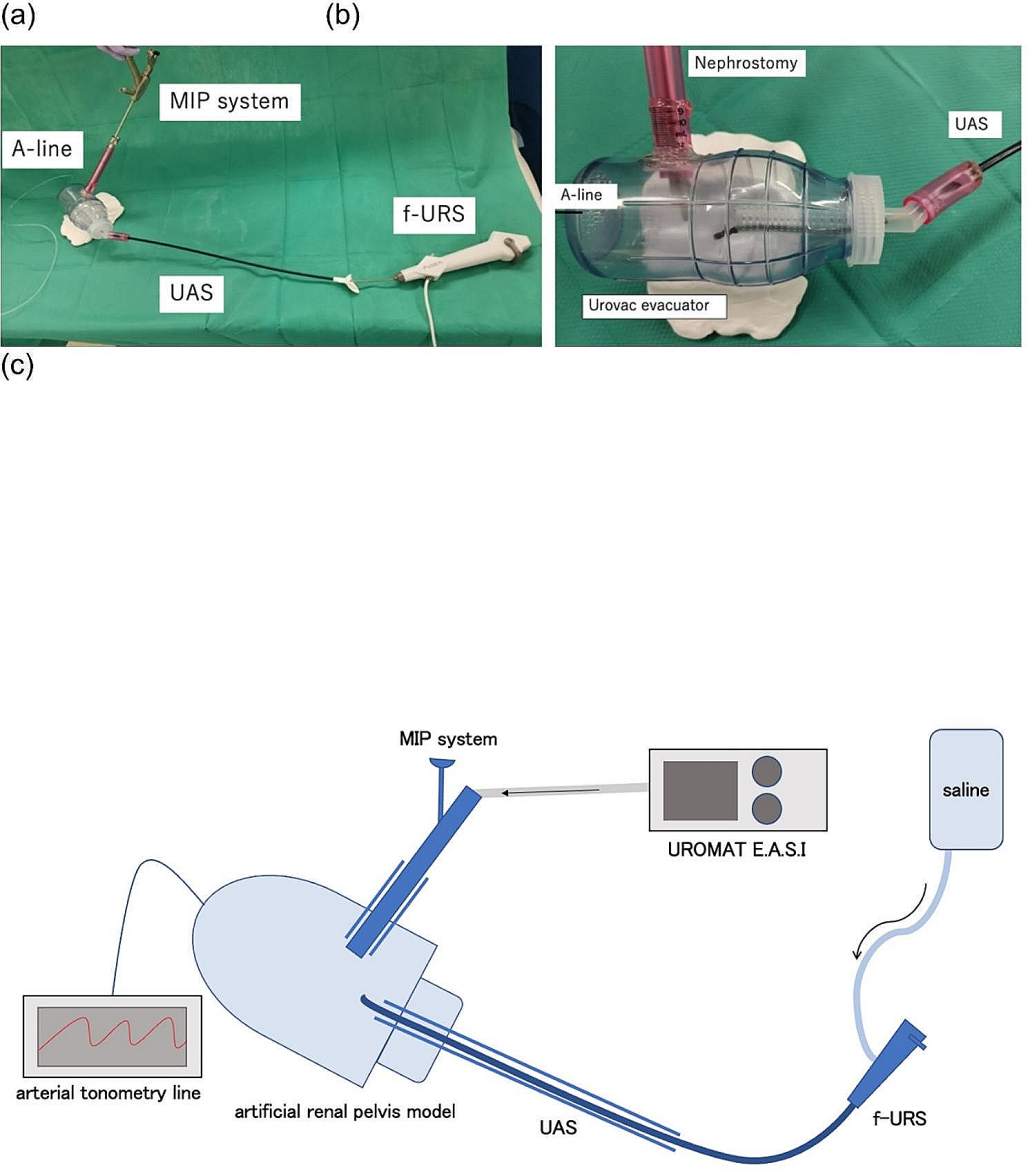
Experimental setup. (**a**) Nephrostomy, UAS, and f-URS placed in the artificial kidney model. The nephrostomy sheath was connected to the Urovac evacuator™ so that it was 70°above the horizontal plane, representing puncture of the lower calyx in a prone position in a human body. The UAS was installed at a site that resembled the renal pelvic ureteral junction. An arterial tonometry line was attached to the head side of it. (**b**) Artificial kidney model details. For nephrostomy and UAS, a silicone connector is used for the connection to the Urovac evacuator™, making it possible to change the sheath size. (**c**) Schematic representation of all equipment used in the experiment. UROMAT E.A.S.I.™ pump was used for irrigation from the nephroscope to achieve a steady continuous irrigation pressure

### Irrigation pressure

We used UROMAT E.A.S.I.™ pump (Karl Storz, Tuttlingen, Germany) for irrigation from the nephroscope to achieve a steady continuous irrigation pressure. It was used to adjust the pressure in four steps: 40 mmHg, 80 mmHg, 120 mmHg and 160 mmHg.

### Flexible ureteroscope

A single-use digital flexible ureteroscope (f-URS) PU3033A (PUSEN Medical, Zhuhai, China) was inserted through a UAS until reaching the renal pelvis. Two types of UAS were used: 10/12 Fr and 12/14 Fr Flexor (Cook Medical, Bloomington, IN, USA). When using the f-URS, the tip was placed in the renal pelvis through the UAS and irrigation pressure was set at 80 cmH_2_O. When not in use, the measurement was performed with the UAS open.

### Nephroscope

For the nephroscope, we used minimally-invasive PCNL (MIP) systems™ (Karl Storz). Four types of MIP systems™ were used, and three types of nephroscope were used according to the sheath system of the outer cylinder: MIP-L (shaft diameter 25/26 Fr) uses 19.5 Fr nephroscope, MIP-M (shaft diameter 16.5/17.5 Fr) uses 12 Fr, MIP-S (shaft diameter 11/12 Fr) and MIP-XS (shaft diameter 8.5/9.5 Fr) uses 7.5 Fr. The working channel was empty.

The length of each sheath and nephroscope was the same, and the insertion position of the nephroscope was aligned in all cases. The tip of the nephroscope was inserted until it protruded 2 cm from the sheath. Irrigation was performed by connecting a UROMAT E.A.S.I. pump™ (Karl Storz) to the nephroscope.

### Test combinations

**Fig. 2:**
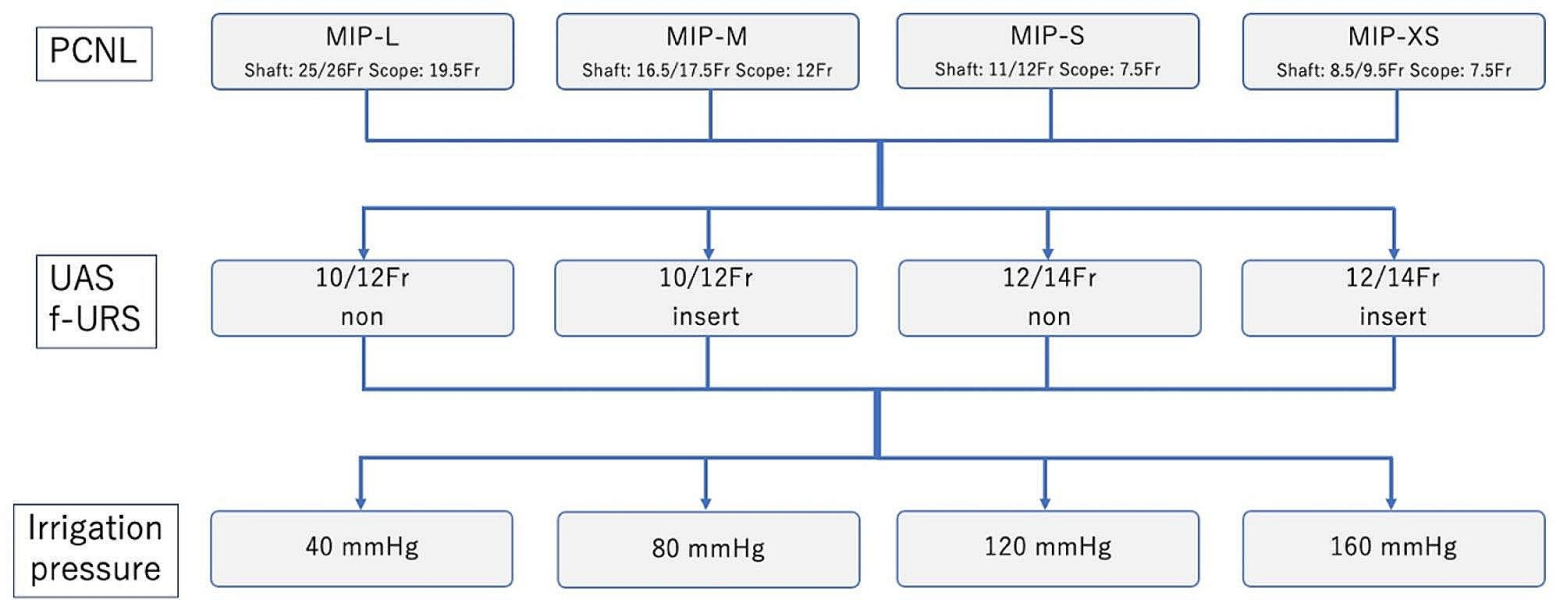
Test combinations. Multiple tests were performed under various combinations of different MIP system™, UAS, f-URS and irrigation pressures. We performed each combination six times

A combination of the MIP system™, UAS and f-URS, adjustment of irrigation pressure from the nephroscope, and multiple combinations were performed. In all experiments, the working channels of both nephroscope and f-URS were empty. All tests were repeated six times for each combination. Test combinations of MIP system™, UAS, f-URS and irrigation pressure are shown in Fig. 2. Zeroing was performed for each test, and after 30 s or more had passed from the start of measurement, it was confirmed that a plateau had occurred, and that value was measured and the average was calculated.

### Statistical analyses

Results were presented as the mean with standard deviation (SD). The Student’s t-test was used for repeated measures in a single group. All trials used JMP PRO version 16.0.0 (JMP, Cary, NC, USA) and *p*-values < 0.05 were considered significant.

## Results

### ECIRS setting (no f-URS inserted); Fig. 3a, b.

In cases of open UAS without f-URS, renal pelvic pressure remained low and never exceeded 30 mmHg, regardless of UAS size or irrigation pressure. Recorded renal pelvic pressure averaged values ranged between 0.2 and 10.0 mmHg. Using 10/12 Fr UAS and MIP-M, the intrapelvic pressure increased most when the irrigation pressure was 160 mmHg, with an average value of 10 mmHg (SD: ± 0).

**Fig. 3:**
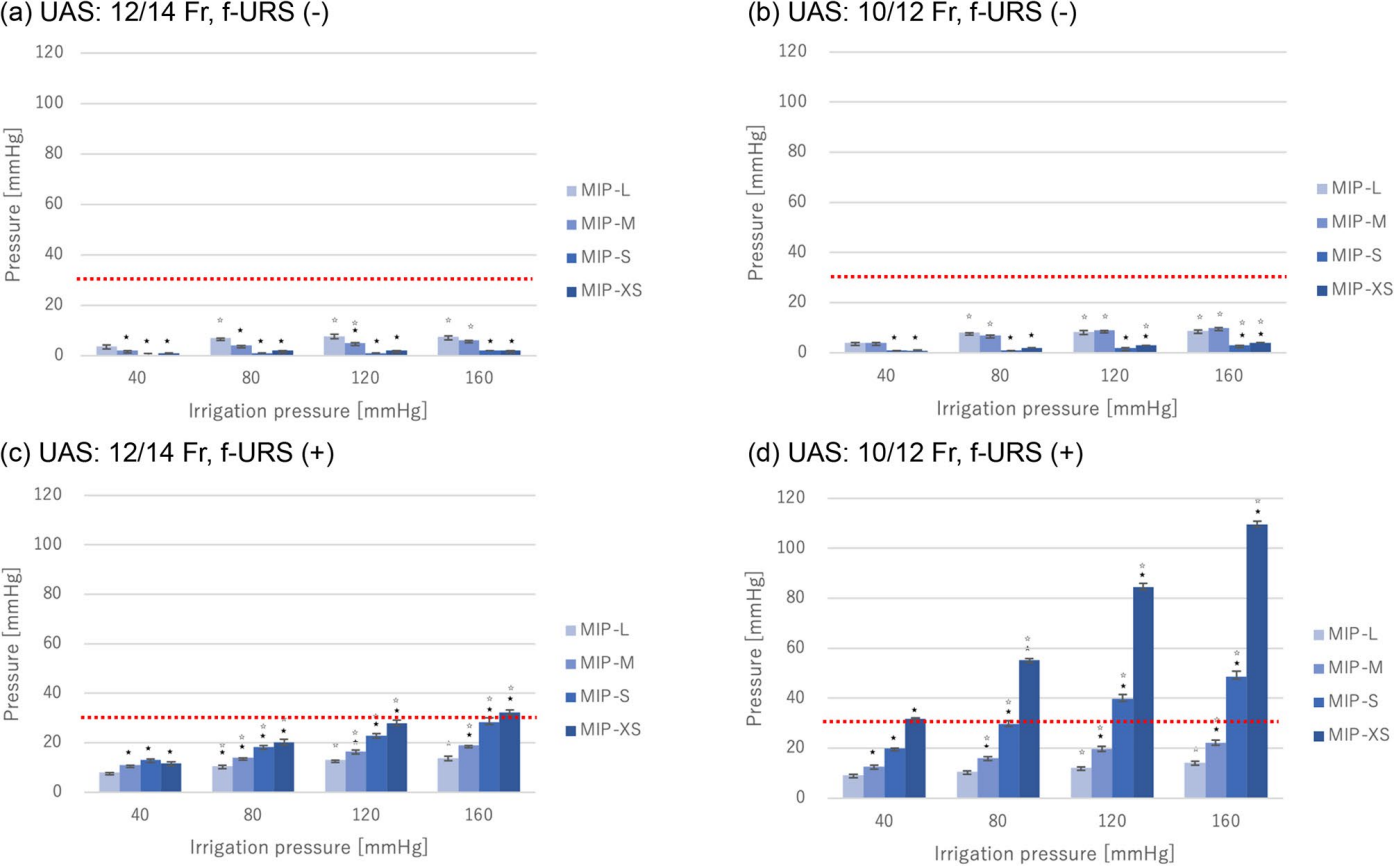
Intrarenal pressure. (**a**) Used 12/14 Fr UAS and f-URS is not inserted. (**b**) Used 10/12 Fr UAS and f-URS is not inserted. (**c**) Used 12/14 Fr UAS and f-URS is inserted. (**d**) Used 10/12 Fr UAS and f-URS is inserted. Values are expressed as mean ± standard deviation. ★Those with *p* < 0.05 when using the value of MIP-L under same irrigation pressure as a reference. ☆Those with *p* < 0.05 when using the value of 40 mmHg with same nephrostomy sheath as a reference

### ECIRS setting (f-URS inserted); Fig. 3c, d.

When a ureteroscope was inserted, the intrarenal pressure increased with decreasing nephroscope size (MIP-L → MIP-M → MIP-S → MIP-XS) and with increasing irrigation pressure (40 → 80 → 120 → 160 mmHg) in all conditions. This trend was more pronounced with narrower UAS (10/12 Fr rather than 12/14 Fr). Specifically, when the UAS was thicker (12/14 Fr), the danger zone of intrarenal pelvic pressure (30 mmHg) was reached only at the maximum irrigation pressure (160 mmHg) using the thinnest nephroscope (MIP-XS). The average intrapelvic pressure under these conditions was 32.3 mmHg (SD: ±1.03) (Fig. 3c).

In contrast, with a narrow UAS (10/12 Fr) and a narrow nephroscope (MIP-XS), the intrarenal pelvic pressures at irrigation pressures of 40, 80, 120, and 160 mmHg were 31.8 mmHg (SD: ±0.41), 55.3 mmHg (SD: ±0.52), 84.5 mmHg (SD: ±1.38), and 109.5 mmHg (SD: ±1.38), all of which exceeded the dangerous range of 30 mmHg. Even with the MIP-S nephroscope, which is one size larger in diameter, the intrarenal pelvic pressures at irrigation pressures of 120 mmHg and 160 mmHg were 39.7 mmHg (SD: ±1.75) and 48.7 mmHg (SD: ±2.25), both of which exceeded the dangerous range of 30 mmHg (Fig. 3d). However, when the diameter of the nephroscope was larger (MIP-M and MIP-L), the pressure in the renal pelvis never exceeded 30 mmHg at any irrigation pressure.

## Discussion

With the evolution of urinary stone treatment equipment and technology, there is a need to evaluate not only efficacy, but also its safety and risks. Studies on renal physiology have shown that intrarenal pressures above 30 mmHg can cause pathological changes in renal tubular histology [14]. Intraoperative renal pelvic pressure is associated with postoperative fever and perioperative sepsis, and research on renal pelvic pressure measurement in endoscopic treatment has become active. Fang et al. measured renal pelvic pressure with f-URS using three sizes of UAS (11/13, 12/14, 13/15 Fr) in vitro in fresh cadaveric porcine urinary systems. In the 11/13 Fr UAS, the renal pelvic pressure exceeded 40 cmH_2_O (29.4 mmHg) when the irrigation pressure exceeded 250 cmH_2_O (183.8 mmHg), and in the 13/15Fr UAS, the renal pelvic pressure exceeded 40 cmH_2_O when the irrigation pressure exceeded 500 cmH_2_O (367.5 mmHg) [15]. Patel et al. measured the renal pelvic pressure in f-URS using different sizes of UAS (12/14 Fr, 14/16 Fr); the thick UAS had significantly lower renal pelvic pressure (25.3 ± 13.1 vs. 44.0 ± 27.5 mmHg, *p* = 0.03). In addition, when the 14/16 Fr UAS was used, the renal pelvic pressure did not exceed the critical level at irrigation pressure of 150 mmHg [16]. These studies featured experiments using f-URS alone, and the model used in the current experiment is different, so there may be differences in the results from these studies. However, it was suggested that the larger the UAS size, the lower the likelihood of increase in renal pelvic pressure. Guohua et al. found average renal pelvic pressures of 24.85, 16.23, and 11.68 mmHg in PCNL with 14, 16, and 18 Fr sheaths, respectively, and generally < 30 mmHg in PCNL with 14–18 Fr percutaneous tracts [17]. The larger the tract size, the larger the gap with the nephroscope, and this tends to lower the renal pelvic pressure. Furthermore, Huusman et al. reported a study of intrarenal pressure with 9.5 Fr and 12 Fr nephroscopes in an ex vivo organ model. In a PCNL experiment using an ultra-thin nephroscope, when a mono-J-catheter was placed in the renal pelvis and suction was applied, the intrapelvic pressure did not exceed 30 mmHg at a normal perfusion pressure [18].

However, intrarenal pelvic pressure in ECIRS has not been widely studied. From the f-URS and PCNL studies, it is expected that the renal pelvic pressure will decrease as the UAS size and percutaneous tract size increase in ECIRS as well. Doizi et al. created an artificial model and used a 15–30 Fr nephroscope sheath and a 10/12 Fr UAS to measure renal pelvic pressure in ECIRS; they reported that the intrapelvic pressure never exceeded 50 cmH_2_0 (36.8 mmHg) [19]. Furthermore, there are no experiments comparing renal pelvic pressure using ultra-thin nephroscopes. This study is the first to measure renal pelvic pressure in ECIRS using an ultra-thin 8.5/9.5 Fr nephroscope using an artificial kidney model. An advantage of ECIRS is that it is possible to operate the nephroscope and f-URS simultaneously. In our study, the intrarenal pelvic pressure could be kept low under any condition if the f-URS was not inserted into the UAS. On the other hand, when the f-URS was inserted, the renal pelvic pressure increased as the nephroscope sheath and UAS became narrower and as the perfusion pressure increased. In particular, the combination of 8.5/9.5 Fr nephroscope sheath and 10/12 Fr UAS should be avoided because the renal pelvic pressure reached a dangerous level (> 30 mmHg) at low irrigation pressure.

Our study has some noteworthy limitations. First, although the artificial kidney model we used has been reported previously [12, 13], we are unsure how similar it is to human tissue in terms of compliance. Animal models might be more suitable for reproducing biological responses and in vivo conditions, but they have the drawbacks of the inability to conduct multiple experiments and they yield unstable results. However, based on our results, the next challenge is to create an experimental model using animals. Second, we did not examine renal pelvic pressure with or without an instrument inserted in the working channel of the nephroscope or f-URS. Doizi et al. reported that inserting a 173 μm laser through the working channel of the nephroscope reduced intrarenal pelvic pressure by up to 9.7 cmH_2_O (7.1 mmHg) [18]. However, in this study, the maximum renal pelvic pressure was 109.5 mmHg when an ultra-thin nephroscope was used, and there is a high possibility of dangerous renal pelvic pressure, regardless of the use of the working channel.

## Conclusions

To maximize the benefits of ECIRS without increasing intrarenal pelvic pressure, care must be taken in the combination of nephroscope sheath and UAS used. This study showed that the use of a f-URS increased intrapelvic pressure in ECIRS using a small or very small PCNL system. Using a thin nephrostomy sheath in ECIRS can lead to increased intrarenal pelvic pressure. Although our study was based on an artificial model, it suggests that special care is required when using a flexible scope simultaneously in treatment of patients.

### Electronic supplementary material

Below is the link to the electronic supplementary material.

Supplementary Material 1

## Data Availability

The datasets analysed during the current study are available from the corresponding author on reasonable request.This document was proofread and edited by Benjamin Phillis at the Clinical Study Support Center at Wakayama Medical University.

## Abbreviations

ECIRS: Endoscopic combined intrarenal surgery
f-URS: Flexible ureteroscope
MIP: Minimally invasive PCNL
PCNL: Percutaneous nephrolithotomy
SD: Standard deviation
UAS: Ureteral access sheath
