# Supplementary material for: Effect of nephrostomy sheath size on renal pelvic pressure during endoscopic combined intrarenal surgery: artificial kidney model study
Source: BMC Urol. 2024 Apr 3;24:77. doi: 10.1186/s12894-024-01458-z (PMC10993431; doi:10.1186/s12894-024-01458-z)
Supplement: Supplementary file 1 — Supplementary Material 1 [file 12894_2024_1458_MOESM1_ESM.docx]

Supplemental table: Intrarenal pressure (mmHg)

| UAS | MIP | Irrigation pressure (mmHg) | | | |
| --- | --- | --- | --- | --- | --- |
|  |  | 40 | 80 | 120 | 160 |
| No f-URS inserted | | | | | |
| 12/14 Fr | MIP-L | 3.7 | 7.0 | 7.0 | 7.3 |
|  | MIP-M | 2.0 | 4.0 | 5.0 | 6.0 |
|  | MIP-S | 0.2 | 1.0 | 1.0 | 2.0 |
|  | MIP-XS | 1.0 | 2.0 | 2.0 | 2.0 |
| 10/12 Fr | MIP-L | 4.0 | 8.0 | 8.3 | 8.8 |
|  | MIP-M | 4.0 | 7.0 | 9.0 | 10.0 |
|  | MIP-S | 1.0 | 1.0 | 2.0 | 3.0 |
|  | MIP-XS | 0.7 | 2 | 3.0 | 4.0 |
| f-URS inserted | | | | | |
| 12/14 Fr | MIP-L | 8.0 | 10.5 | 13.0 | 13.8 |
|  | MIP-M | 11.0 | 14.0 | 16.5 | 19.0 |
|  | MIP-S | 13.2 | 18.3 | 23.0 | 28.3 |
|  | MIP-XS | 11.8 | 20.2 | 27.8 | 32.3 |
| 10/12 Fr | MIP-L | 9.2 | 10.5 | 12.2 | 14.3 |
|  | MIP-M | 12.7 | 16.0 | 19.7 | 22.2 |
|  | MIP-S | 20.0 | 29.7 | 39.7 | 48.7 |
|  | MIP-XS | 31.8 | 55.3 | 84.5 | 109.5 |
